# Post-treatment changes in hematological parameters predict response to nivolumab monotherapy in non-small cell lung cancer patients

**DOI:** 10.1371/journal.pone.0197743

**Published:** 2018-10-25

**Authors:** Monica Khunger, Pradnya Dinkar Patil, Arjun Khunger, Manshi Li, Bo Hu, Sagar Rakshit, Arnab Basu, Nathan Pennell, James P. Stevenson, Paul Elson, Tanmay S. Panchabhai, Vamsidhar Velcheti

**Affiliations:** 1 Department of Internal Medicine, Cleveland Clinic, Cleveland, Ohio, United States of America; 2 Department of Hematology and Oncology, Taussig Cancer Institute, Cleveland Clinic, Cleveland, Ohio, United States of America; 3 Department of Quantitate Health Sciences, Cleveland Clinic Foundation, Cleveland, Ohio, United States of America; 4 Department of Hematology and Oncology, University of Southern California, Los Angeles, California, United States of America; 5 Norton Thoracic Institute, St. Joseph’s Hospital and Medical Center, Phoenix, Arizona, United States of America; Baylor College of Medicine, UNITED STATES

## Abstract

**Background:**

The absolute neutrophil count (ANC), absolute lymphocyte count (ALC), absolute monocyte count (AMC) and neutrophil to lymphocyte ratio (NLR) are known markers of inflammation. We evaluated whether ANC, ALC, AMC and NLR, both before and after treatment with nivolumab, are indicative markers of overall survival (OS) and evaluated change in NLR as a predictive marker of response in non -small cell lung cancer (NSCLC) patients treated with nivolumab.

**Methods:**

A total of 109 patients with advanced NSCLC treated with nivolumab were included. ANC, ALC, AMC and NLR were examined at initiation of nivolumab therapy and after two cycles. The prognostic role of ANC, ALC, AMC and NLR with OS and changes in NLR ratio were examined with Kaplan-Meier curves and proportional hazard model.

**Result:**

Post-treatment NLR ≥5 after two cycles of nivolumab was associated with poor OS (median OS in NLR = <5 vs NLR = ≥5 was 29.1 (16.2–40.9) vs 24.2(16.1–36.2) months respectively, p<0.001). In addition NLR increased in non-responders after two cycles of nivolumab by 6.6±21.8 as compared to responders (p = 0.027).

**Conclusions:**

Post-treatment ANC, ALC and NLR are independent prognostic factors in NSCLC patients treated with nivolumab. Changes in NLR can be an early biomarker for response in NSCLC patients treated with nivolumab.

## Introduction

Lung cancer is the most common cause of cancer related death in the United States and worldwide [1, 2]. An estimated 80–85% of patients with lung cancer have non-small-cell lung cancer (NSCLC). The recent success of immune checkpoint inhibitors in the ability to achieve durable responses in patients with NSCLC with a relatively well tolerated side effect profile has resulted in a paradigm shift in the treatment of patients with advanced NSCLC. Currently, two PD-1(programmed death -1) inhibitors, nivolumab and pembrolizumab and one PD-L1 (programmed death ligand -1) inhibitor, atezolizumab have been approved by the Food and Drug Administration (FDA) for treating patients with advanced NSCLC who have progressed after chemotherapy. In addition, pembrolizumab is approved in the front-line setting for advanced NSCLC patients as monotherapy (for PD-L1 expression greater than 50%) or in combination with platinum based chemotherapy (regardless of PD-L1 expression). However, the response rate to immunotherapy is quite modest and there is a lack of biomarkers to help distinguish responders from non-responders. Many studies have tried to explore the role of PD-L1 expression within the tumor as a biomarker, however these studies have noted that patients respond to PD-1/PD-L1 inhibitors despite “negative” PD-L1 expression. [3, 4] This observation was most prominent in patients with advanced squamous cell lung cancer treated with nivolumab. [4] Subsequently, several other trials have tried to explore TILs (tumor infiltrating lymphocytes), tumor mutational load and IL-8 as biomarkers, often with mixed results. [5–9]

Systemic inflammation has been linked to poor outcomes in many types of solid tumors. Inflammation has been associated with both the development and progression of cancer. [10] The presence of tumor associated neutrophils [11–13] macrophages [14, 15] and platelets [16, 17] in the tumor microenvironment have been shown to promote tumor growth and aide metastatic spread, therefore resulting in poor outcomes in a variety of malignancies. Tumor infiltrating lymphocytes, on the other hand have been associated with better outcomes in cancer patients including those with NSCLC. [18–24] Peripheral hematologic parameters such as absolute neutrophil count (ANC), absolute lumphocyte count (ALC), neutrophil to lymphocyte ratio(NLR) and absolute monocyte count (AMC) serve as surrogate markers of inflammation in the host and may be reflective of inflammation in the tumor microenvironment. While the exact relationship between tumor infiltrating cells (TILs) and circulating hematologic cells remains to be explored; a recent study by Dirican et al demonstrated a correlation between the TILs in the tissue microarrays of patients with NSCLC and NLR. [25]The study showed negative correlation between intratumoral CD3+ TILs and NLR and positive correlation between intratumoral CD5+ TILs and NLR. Further high intratumoral CD3+ and low CD5+ were associated with poor OS. Bagley et al concluded that high pre-treatment NLR was associated with poor OS in nivolumab treated NSCLC patients. [26] However ours is the first study to date exploring post-treatment changes in NLR ratio amongst responders and non-responders to establish the role of NLR as a predictive biomarker of durable clinical benefit with nivolumab.

## Methods and materials

### Patient selection

**Fig 1:**
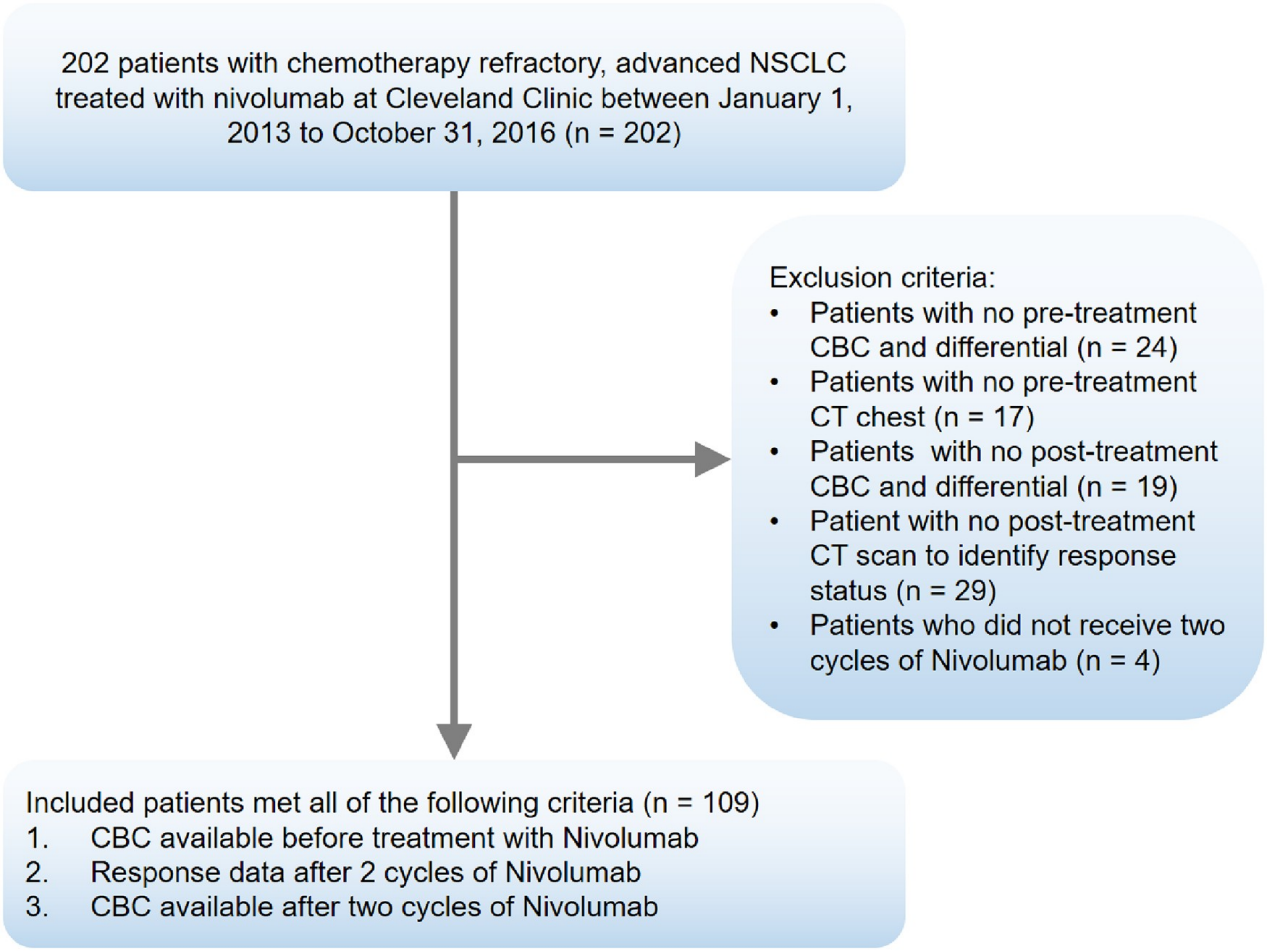
Schema of patient inclusion criteria.

The study was approved by the Cleveland Clinic Institutional Review Board (IRB) and the requirement for informed consent was waived for this study by the IRB. Clinicopathologic data for all patients with advanced NSCLC treated with nivolumab between January 1, 2013 and October 31, 2016 at Cleveland Clinic was obtained by reviewing their electronic medical records. All data was organized in the REDCap^™^ database. We exclusively focused on patients treated with nivolumab (and not other PD-1/PD-L1 inhibitors) due to treatment practices at our institution as well as the fact the maximum data was available for nivolumab treated patients as compared to other treatments. This further helped minimize heterogeneity related to treatment with different PD-1/PD-L1 inhibitors. Data regarding patient demographics, histologic classification, tumor grade, line of treatment of nivolumab, driver mutation, complete blood count including a differential leukocyte count prior to treatment and after two cycles of nivolumab were collected. The RECIST (Response Evaluation Criteria in Solid Tumors) 1.1 criteria were used to identify tumor response after completing two cycles of therapy with nivolumab. Fig 1 represents schema of patient inclusion criteria.

**Fig 2:**
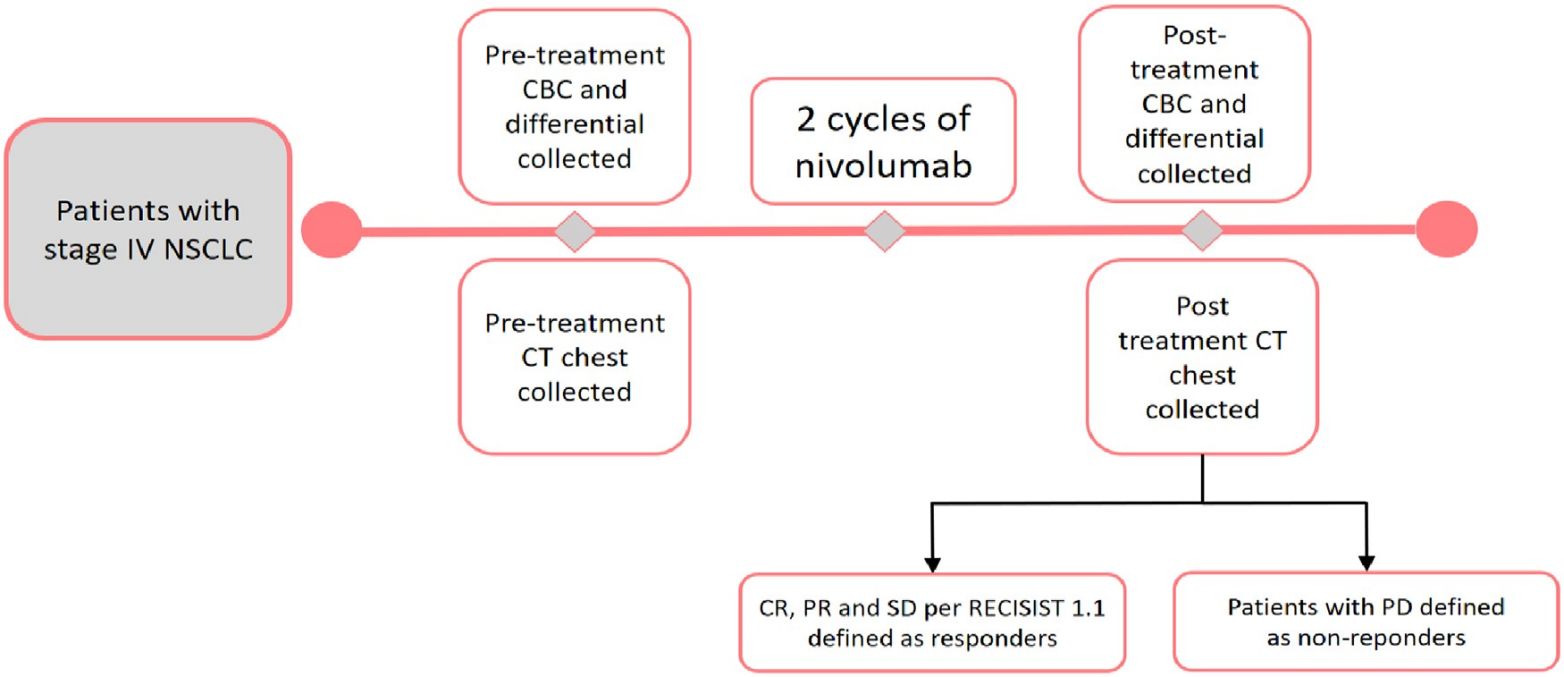
Study timeline.

The primary exposure variables were pre-treatment and post-treatment ANC, ALC, AMC and NLR. Pre-treatment and post-treatment NLR were calculated as the ratio of pre-treatment and post-treatment ANC to ALC respectively. Fig 2 represents the timeline of the study including the pre-treatment CBC, pre-treatment CT chest, post-treatment CBC and post-treatment CT chest collection for assessment of response. Subgroup analysis were performed for the following pre-determined co-variables: sex of the patient, line of nivolumab therapy (<3^rd^ line vs ≥ 3^rd^ line and beyond), smoking status (current, former and never smoker), presence of a targetable oncogenic driver mutation (defined as having *EGFR* mutation and *ALK* translocation) and histology (squamous vs. adenocarcinoma). Data for tumor PD-L1 status was not available for majority of the patients and hence was not included in the analysis.

#### Cut-off selection of hematological parameters

Patients were dichotomized based on a cutoff value of NLR ≥ 5 and <5, as NLR ≥ 5 has been previously associated with inferior overall survival (OS) and progression free survival in melanoma patients treated with ipilimumab and NSCLC patients. [26, 27]Overall survival (OS) was the primary outcome, defined as the duration in months between the first dose of nivolumab and death. The secondary outcome of interest was durable clinical benefit (patients with complete response, partial response and stable disease) after two cycles of nivolumab as per RECIST v1.1.

### Statistical analysis

Statistical analyses were performed using SAS 9.3 (Cary, NC) and Stata 14.1 (Stata, College Station, TX). A recursive partioning algorithm was used to define the cutoffs for each of the inflammatory indices. ANC and ALC were divided into four quartiles as described previously; an elevated NLR was defined as ≥5. [26] The clinicopathological variables were sumamrized as appropriate descriptive statistics. Survival analysis was used to identify factors associated with poor OS and Kaplan meier curves were plotted for categories of pre-treatment and post-treatment ANC, ALC, AMC and NLR. Log-rank test was used to compare the survival curves. A multivariate proportional hazard model was also fitted to the data with the assumption of proportional hazard verified with residual plots. Patients were further divided into groups with durable clinical benefit (complete response, partial response and stable disease) and non-responders (progressive disease) based on their response per RECIST v1.1 to nivolumab after 2 cycles of therapy. The change in absolute NLR after 2 cycles of nivolumab in the two groups was studied using the Wilcoxon rank sum test. Statistical significance was esablished with a two-sided p-value<0.05.

## Results

A total of 109 patients with advanced NSCLC treated with nivolumab were included. Median age at diagnosis was 67 (range 45–90) years. Median OS of the included patients treated with nivolumab was 25 months. Median baseline ANC and ALC (IQR) were 5.3(3.7–6.8) and 1.07 (0.77–1.4) respectively. Fifty percent of the entire patient population had NLR <5 while the rest half had NLR ≥ 5 at baseline. All included patients had ECOG performance status of 0-II. The vast majority of the cohort (84%) consisted of current or former smokers and seventy six percent patients had non-squamous histology. Ninety one percent patients had received one prior systemic therapy and in fifty percent of the patients, nivolumab was second line of treatment after progression from front-line chemotherapy. Table 1 summarizes the baseline characteristics of all included patients.

**Table 1:** Baseline characteristics of all included patients.

Variable	N (%) or Median (Range)
Sex	
Male	56(51.4%)
Female	53(48.6%)
Age (mean) at Start of nivolumab	67 (45–90)
Histology	
Adenocarcinoma	71(65.2%)
Squamous cell	26 (23.8%)
Other	12(11%)
Smoking History	
Current	14(12.8%)
Former	78(71.6%)
Never	17(15.6%)
eGFR mutation	
Present	10(9%)
Absent	86(79%)
Unknown	13(12%)
ALK mutation	
Present	0
Absent	93(85%)
Unknown	16 (15%)
Pre-treatment ANC	
Min—3.72	28(25.7%)
3.72–5.29	27(24.8%)
5.29–6.78	27(24.8%)
6.78—Max	27(24.8%)
Pre-treatment ALC	
Min—0.77	27(24.8%)
0.77–1.07	27(24.8%)
1.07–1.40	27(24.8%)
1.40—Max	28(25.6%)
Pre NLR	
<5	54(49.5)
≥5	55(50.5)
Post NLR	
<5	58(53.2)
≥5	51(46.8)
Pre AMC	
<0.5	26 (23.9%)
0.5–0.8	40(36.7%)
≥0.8	43(39.4%)
Median interval between diagnosis and treatment with Nivolumab	8.5 months
Median OS (95% CI)	25.1(15.6–39) months
Nivolumab Treatment “Line”	
First line	10(9.2)
Second line	51(46.8)
Third line	23(21.1)
Fourth line	17(15.6)
Fifth line and beyond	8(7.3)
No. Doses Given	9 (1–31)
No. of deaths	65

### Prognostic role of hematological parameters

**Fig 3:**
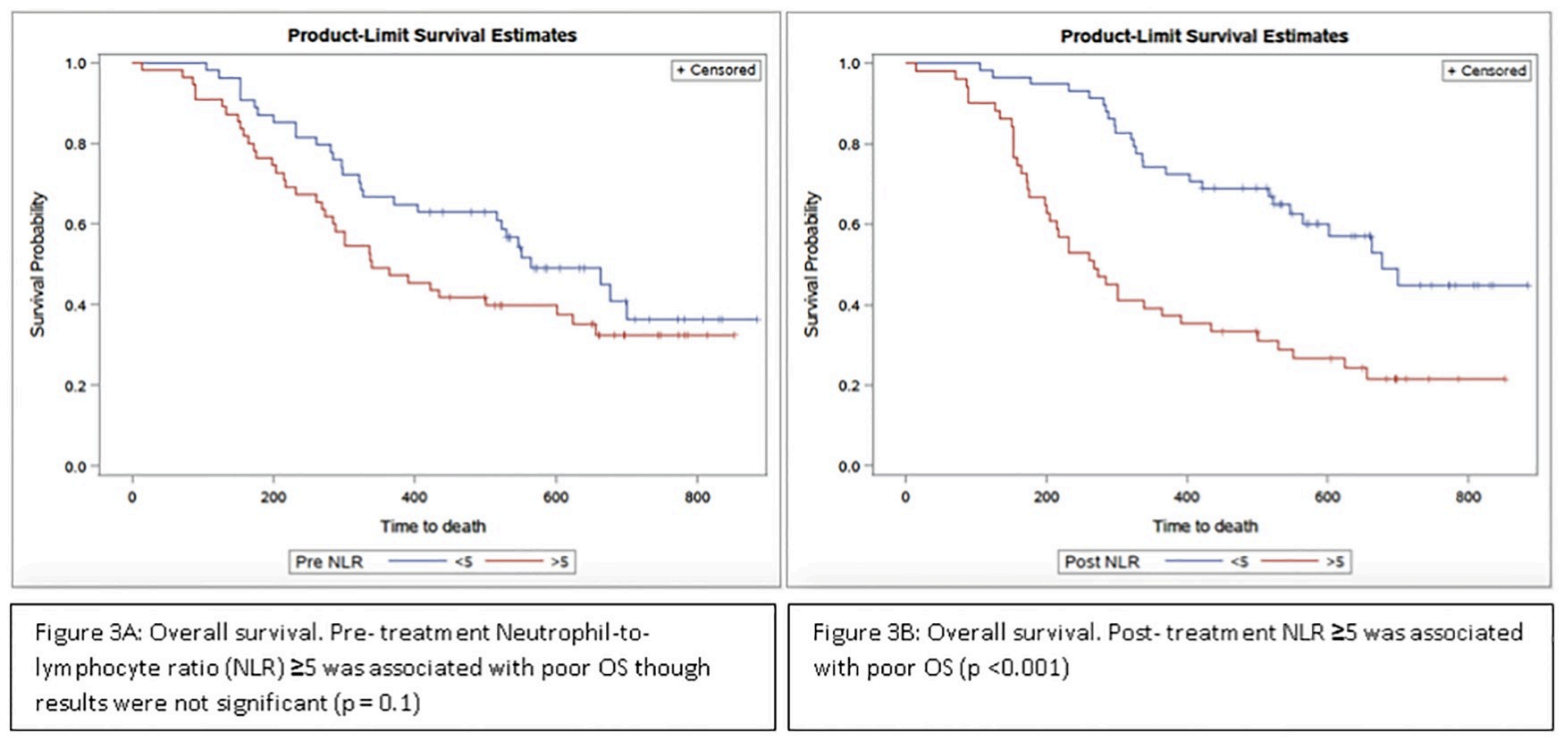
Kaplan–Meier curve representing association of OS with pre-treatment NLR (fig 3A) and post-treatment NLR (fig 3B).

The median duration of follow up was 30 months. Pre-treatment NLR ≥5 was associated with inferior OS as compared to NLR<5, however the difference was not statistically significant (median OS in months NLR <5 vs. NLR ≥5 months was 26.4(13.6–40.9) and 25.8(17.3–34.6) respectively; p = 0.1, Fig 3A). Post-treatment NLR ≥5 was statistically significantly associated with inferior OS (median OS in NLR = <5 vs. NLR = ≥5 was 29.1 (16.2–40.9) vs 24.2(16.1–36.2) months respectively, p<0.001; Fig 3B.

**Fig 4:**
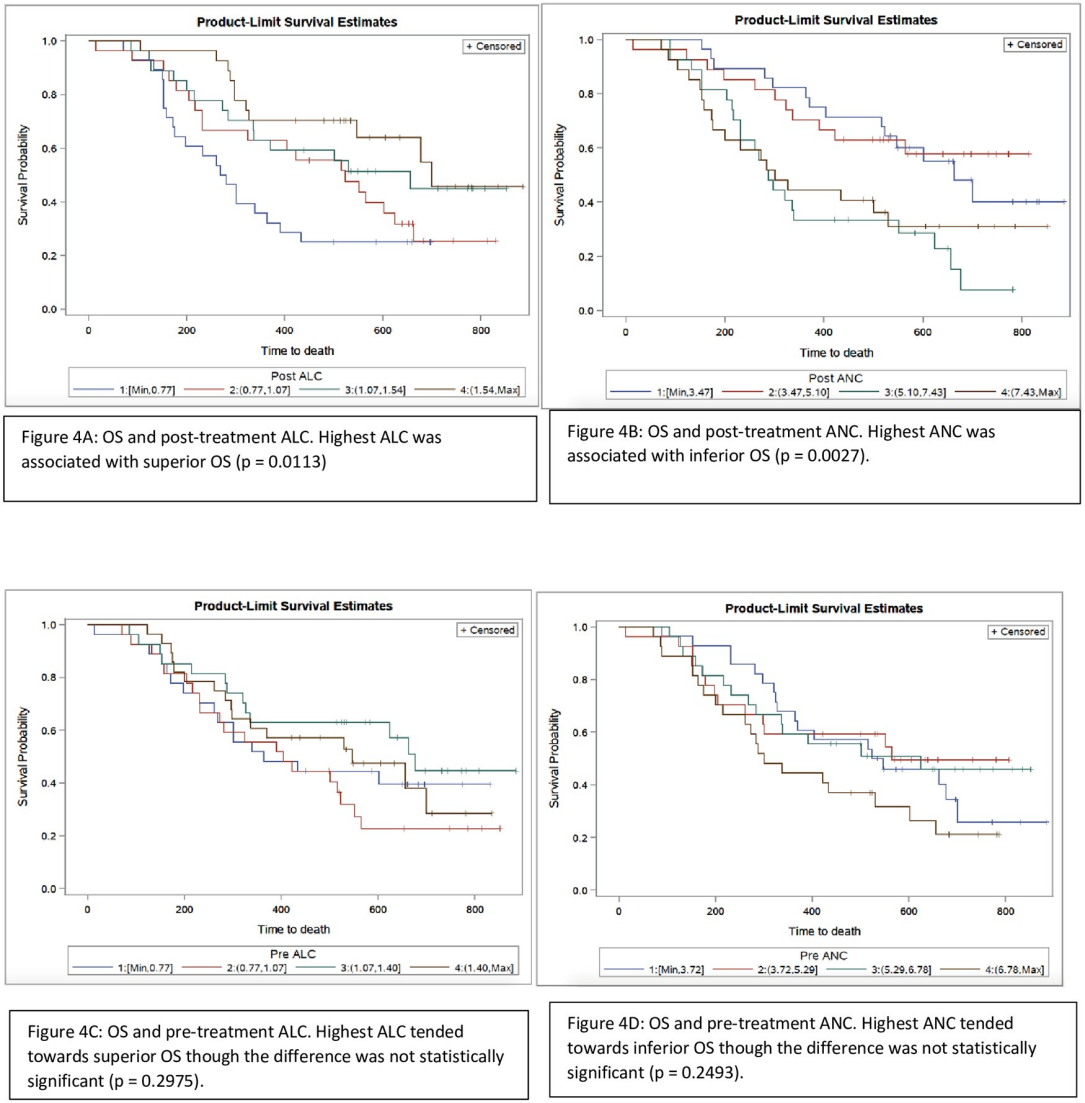
Kaplan–Meier curve representing association of OS with post-treatment ALC (fig 4A), post-treatment ANC (fig 4B), pre-treatment ALC (fig 4C) and pre-treatment ANC (fig 4D).

We performed an exploratory analysis to determine whether differences in OS and pre- and post-treatment NLR were attributable mainly to the ALC, ANC, or both. We divided both pre and post-treatment ANC and ALC cohorts into quartiles. We generated survival curves by quartile for each of these values according to the Kaplan-Meier method. Patients in the highest quartile of post-treatment ALC had superior OS compared to the rest of the population (log-rank p = 0.0113; Fig 4A). Patients in the highest quartile of post-treatment ANC had inferior OS compared to all others (log-rank p = 0.0027; Fig 4B). Patients in the highest quartile of pre-treatment ALC tended towards superior OS however the result was not statistically significant (p = 0.2975; Fig 4C). Similarly patients in the highest quartile of pre-treatment ANC tended towards inferior OS as compared to all other patients, however the results were not statistically significant (p = 0.2493; Fig 4D). No significant difference between pre-treatment and post-treatment AMC with OS were noted. (S1 Fig).

### Predictive role of hematological parameters

Non-responders had an increase in NLR by 6.6±21.8, whereas patients with stable disease, partial or complete response after two cycles of nivolumab were noted to have an increase in NLR by only 0.25±8.6 and this difference was statistically significant (p = 0.037). Table 2 summarizes the mean ANC, ALC, AMC and NLR in patients with advanced NSCLC, both pre-treatment and after two cycles of nivolumab. Patients who had progressive disease after two cycles of nivolumab were found to have statistically significantly higher AMC as compared to responders. However no significant differences in the mean ANC, ALC and NLR were noted among responders and non-responders.

**Table 2:** Summary statistics for hematological parameter among patients with objective response (CR, PR) as compared to non-responders (PD).

Hematological parameter	Mean pre-treatment hematological parameter in responders	Mean pre-treatment hematological parameter in non-responders	P value of pre-treatment hematological parameter among responders and non-responders	Mean post-treatment hematological parameter in responders	Mean post-treatment hematological parameter in non- responders	P value of post-treatment hematological parameter among responders and non-responders
ANC	5.4±2.5	6.3±3.3	0.13	7.6±12.8	10.3±16.0	0.39
ALC	1.2±0.58	1.2±0.64	0.77	1.8±2.4	1.1±0.69	0.070
NLR	6.1±4.4	6.5±3.9	0.66	6.6±11.0	13.1±22.4	0.093
AMC	0.73±0.33	0.75±0.34	0.85	0.63±0.24	0.76±0.34	***0*.*035***

Multivariate Cox proportional hazards model was used to determine whether pre and post treatment ANC, ALC, AMC and NLR and/or other baseline characteristics were associated with OS. Post treatment ANC and ALC were significantly associated with OS using the multivariate Cox proportional hazards model. High Post treatment ANC ≥5.10 as compared to patients with ANC <5.10 was associated with inferior OS (HR = 4.7, 95% CI, 1.5–15.1; p = 0.0087). On the contrary, high post treatment ALC ≥0.77 was associated with superior OS as compared to patients with low post-treatment ALC (HR = 0.36, 95% CI, 0.15–0.86; p = 0.02). However in the multivariate Cox proportional hazards model, no significant changes in pre or post treatment AMC were observed.

Sub-group analysis of pre-treatment as well as post-treatment NLR, ANC, AMC and ALC was performed based on tumor histology. No significant differences in the pre-treatment NLR and OS were noted in patients with squamous cell lung carcinoma (n = 26) and adenocarcinoma (n = 71; p = 0.4143 and p = 0.6980 respectively).

The median duration between diagnosis and treatment of nivolumab was 8.5 months. Among patients who were treated with nivolumab early during their disease course, defined as time duration between diagnosis and nivolumab treatment < 8.5 months (n = 54), the pre-treatment NLR was 6.6±4.4. Among patients who were treated with nivolumab ≥ 8.5 months after diagnosis (n = 54), the pre-treatment NLR was 5.7±3.9 and the difference between the two groups was not statistically significant; p = 0.26. Similarly, no significant differences between NLR were noted in patients who received nivolumab as <3^rd^ line treatment (n = 74 patients) as compared to patients who received nivolumab as ≥3^rd^ line of treatment (n = 25; 6.3±4.2 vs 5.6±3.7 respectively, p = 0.48).

## Discussion

Our present study included 109 patients with advanced NSCLC treated with nivolumab and correlated pre and post treatment hematological parameters with OS. Post-treatment NLR ≥5 and high post-treatment ANC were associated with worse OS. High ALC was associated with superior OS in NSCLC patients treated with nivolumab. In addition patients with progressive disease after nivolumab treatment had a statistically significant increase in NLR as compared to patients with durable clinical benefit and the differences were statistically significant. This observation suggests that changes in NLR ratio as well as post-treatment ANC and ALC can be a potential predictive biomarker for response in NSCLC patients treated with nivolumab.

Tumor cells secrete chemokines such as IL-8 and recruit neutrophils into the tumor which in return promote angiogenesis, result in growth factor release, and aide vascular invasion, therefore increasing metastatic potential. Subsequently, numerous studies have shown that tumor associated neutrophils are associated with poor outcomes in many malignancies. [11–13] PD-1/PD-L1 inhibitors decrease anti-tumor immune tolerance and subsequently increase anti-tumor immunity by blocking negative regulators of T cells. Hence it is theoretically plausible that treatment response could produce relative alterations in the proportions of circulating neutrophils and lymphocytes and vice versa.

PD-L1 testing has its limitations and while the utility of TILs, tumor mutation burden as biomarkers is currently being explored; complete blood count and differential testing is inexpensive and routinely performed every cycle in patients treated with PD-1/PD-L1 inhibitors making NLR an inexpensive, universally accessible predictive and prognostic marker of response to therapy.

Our study had several limitations due to its retrospective study design. The presence of concurrent inflammatory states or use of immunomodulatory drugs which could have influenced the inflammatory markers could not be ruled out. It is a single center study and further prospective, multi-institution studies are warranted to investigate the utility of these markers in clinical practice.

## Conclusion

Our study shows that an elevated NLR after two cycles of nivolumab as well as high ANC after treatment was significantly associated with worse OS in patients with advanced NSCLC. On the contrary high ALC post-treatment was associated with superior outcomes in NSCLC. An increase in NLR after two cycles was seen in patients who had disease progression. However, further multi-institutional studies are warranted to establish NLR as a prognostic biomarker and changes in NLR as a predictive marker of response to PD-1/PD-L1 inhibitors.

## Supporting information

S1 Fig Kaplan–Meier curve representing association of OS with pre-treatment AMC (supplementary Fig 1A) and post-treatment AMC (supplementary Fig 1B). (TIF)
