# Supplementary figures and images for: Post-treatment changes in hematological parameters predict response to nivolumab monotherapy in non-small cell lung cancer patients
Source: PLoS One. 2018 Oct 25;13(10):e0197743. doi: 10.1371/journal.pone.0197743 (PMC6201866; doi:10.1371/journal.pone.0197743)

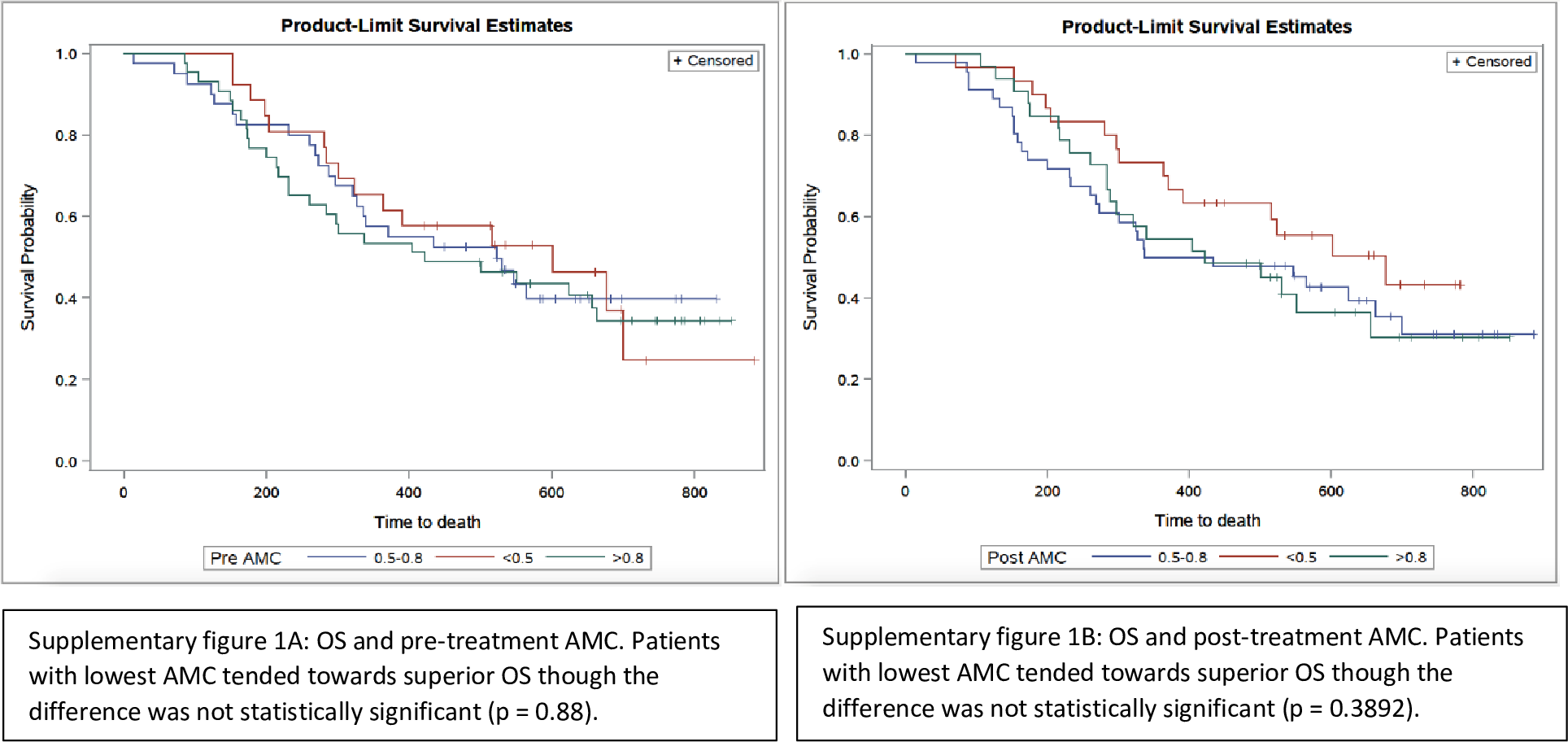

Supplement: S1 Fig — (TIF) [file pone.0197743.s001.tif]
